# Andrew Mathias

**Published:** 1823-08

**Authors:** 


On Wednesday, 16th July, ANDREW MATHIAS, Esq. Surgeon to the General Lying-in Hospital. This gentleman was well and favourably known to the profession by his work on the "Mercurial Disease," which has passed through several editions. Mr. M.'s death was the consequence of an apoplectic fit, but he had never thoroughly recovered from the effects of a severe accident which he met with last year.

